# A Culturally Tailored Artificial Intelligence Chatbot (K-Bot) to Promote Human Papillomavirus Vaccination Among Korean Americans: Development and Usability Study

**DOI:** 10.2196/71865

**Published:** 2025-04-07

**Authors:** Minjin Kim, Ellie Kim, Hyeongsuk Lee, Meihua Piao, Brittany Rosen, Jeroan J Allison, Adrian H Zai, Hoa L Nguyen, Dong-Soo Shin, Jessica A Kahn

**Affiliations:** 1 College of Nursing University of Cincinnati Cincinnati, OH United States; 2 Department of Population and Quantitative Health Sciences University of Massachusetts Chan Medical School Worcester, MA United States; 3 College of Medicine University of Cincinnati Cincinnati, OH United States; 4 Research Institute of AI and Nursing Science College of Nursing Gachon University Incheon Republic of Korea; 5 School of Nursing Chinese Academy of Medical Sciences Chinese Academy of Medical Sciences & Peking Union Medical College Beijing China; 6 Division of Adolescent and Transition Medicine Cincinnati Children's Hospital Medical Center Cincinnati, OH United States; 7 Department of Population and Quantitative Health Sciences Division of Health Informatics and Implementation Science University of Massachusetts Chan Medical School Worcester, MA United States; 8 Department of Population and Quantitative Health Sciences Division of Epidemiology University of Massachusetts Chan Medical School Worcester, MA United States; 9 School of Nursing Hallym University Chuncheon Republic of Korea; 10 Department of Pediatrics Albert Einstein College of Medicine Bronx, NY United States

**Keywords:** human papillomavirus, HPV vaccination, artificial intelligence, AI, chatbot intervention, Korean Americans, usability testing, culturally tailored intervention

## Abstract

**Background:**

Human papillomavirus (HPV) is the most common sexually transmitted infection (STI) worldwide and is associated with various cancers, including cervical and oropharyngeal cancers. Despite the availability of effective vaccines, significant disparities in HPV vaccination rates persist, particularly among racial and ethnic minorities, such as Korean Americans. Cultural stigma, language barriers, and limited access to tailored health information contribute to these disparities.

**Objective:**

This study aimed to develop and evaluate the usability of K-Bot, an artificial intelligence (AI)–powered, culturally tailored, bilingual (Korean and English) chatbot designed to provide culturally sensitive health information about HPV vaccination to Korean immigrants and Korean Americans.

**Methods:**

K-Bot was developed using CloudTuring and Google Dialogflow. Its dialogues were created using Centers for Disease Control and Prevention (CDC) evidence-based HPV information and tailored to the Korean American population based on findings from previous studies. The evaluation and refinement process for K-Bot was organized into 3 phases: (1) expert evaluation by a multidisciplinary panel, (2) usability testing, and (3) iterative refinement based on feedback. An online survey collected demographics, HPV awareness, and vaccination status before 6 focus groups (N=21) sessions using semistructured questions guided by Peter Morville’s usability framework. Quantitative data were analyzed descriptively, and thematic analysis assessed usability, cultural relevance, and content clarity across 6 dimensions: desirability, accessibility, findability, credibility, usability, and usefulness.

**Results:**

Participants had a mean age of 23.7 (SD 4.7) years, with most being female (n=12, 57.1%), second-generation individuals (n=13, 61.9%), and single (n=20, 95.2%). HPV awareness was high (n=19, 90.5%), vaccine knowledge was also high (n=18, 81.8%), but only 11 (52.4%) participants were vaccinated. Feedback-driven refinements addressed usability challenges, including simplifying navigation and adding visual elements. Participants described K-Bot as a promising tool for promoting HPV vaccination among Korean and Korean American users, citing its bilingual functionality and culturally tailored content as key strengths. Evidence-based information was valued, but participants recommended visuals to improve engagement and reduce cognitive load. Accessibility concerns included broken links, and participants proposed enhancements, such as animations, demographic-specific resources, and interactive features, to improve usability and engagement further.

**Conclusions:**

Usability testing of K-Bot revealed its potential as a culturally tailored, bilingual tool for promoting HPV vaccination among Korean immigrants and Korean Americans. Participants valued its evidence-based information, cultural relevance, and bilingual functionality but recommended improvements, such as enhanced navigation, visual elements, and interactive features, to boost engagement and usability. These findings support the potential of AI-driven tools to improve health care access by addressing key barriers to care. Further research is needed to evaluate their broader impact and optimize their design and implementation for individuals with diverse health care needs.

## Introduction

Human papillomavirus (HPV) is the most common sexually transmitted infection (STI) worldwide, with the highest prevalence observed among young adults [1]. Persistent HPV infection can lead to various cancers, including cervical cancer, which remains a leading cause of cancer-related deaths among women aged 20-39 years [2]. Additionally, HPV is associated with oropharyngeal cancers, which are increasingly prevalent among men and tend to develop at younger ages compared to other HPV-related cancers [3].

To combat the burden of HPV-related diseases, the US Advisory Committee on Immunization Practices (ACIP) recommends routine HPV vaccination for adolescents aged 11-12 years, with catch-up vaccination for individuals aged 13-26 years who have not completed the vaccine series [4]. Recently, the 9-valent vaccine was approved in the United States for use in adults aged 27-45 years [5]. Korean Americans are at a relatively high risk for HPV-related cancers, yet they exhibit significant disparities in HPV vaccination rates [6,7]. Notably, Korean American women demonstrate a particularly low intention to receive the HPV vaccine, with only 34.6% expressing a willingness to get the vaccine in prior studies [8]. Our previous research further indicates that only 32% of participants aged 27-45 years have received the HPV vaccine at least once, with differences by sex (females: 35.3%; males: 12.5%) [9]. Korean Americans face several critical barriers to HPV vaccination, including cultural stigma surrounding STIs, language barriers, and al ack of culturally tailored health information. These challenges are compounded by limited knowledge and awareness of HPV and the benefits of vaccination, contributing to persistently low vaccination uptake, which contributes to lower vaccination rates [8-12]. In addition to this knowledge gap, several factors influence HPV vaccination intentions among Koreans and Korean Americans. These include parental recommendations for the HPV vaccine, the perceived benefits and severity of HPV infection, a history of cervical cancer, beliefs about cervical cancer screening for daughters, experiences with sexual activity, and sociodemographic factors (eg, occupation, education, and income) [7,8]. To address these challenges, culturally tailored interventions are essential to improve HPV vaccination uptake within this population, especially for communities that may encounter cultural and linguistic challenges in accessing health care information. For example, prior efforts have included culturally relevant health information that has demonstrated promise in increasing awareness and promoting vaccination by addressing cultural stigmas and misconceptions [9,13,14]. However, many of these approaches rely on traditional methods of information dissemination, which may lack the interactivity and personalization needed to address individual barriers effectively.

KakaoTalk is the most popular mobile messaging app in South Korea. It has become an integral part of daily communication and workplace interaction, offering various features beyond basic text messaging [10]. KakaoTalk has also been used in health research, demonstrating its potential as a platform for delivering health interventions. Studies have explored its role in supporting Korean American family caregivers [11] and promoting health interventions to Korean Americans with limited English proficiency [8], including efforts to promote HPV vaccination [12]. Additionally, KakaoTalk serves as a digital communal space for middle-aged Korean women [15], demonstrating its broad accessibility and significance across diverse Korean populations, from teenagers to older adults, both in South Korea and among Korean Americans.

Artificial intelligence (AI) chatbots have emerged as innovative tools for delivering personalized, leveraging advanced capabilities to address barriers in health communication [16]. Unlike static interventions, such as printed materials or one-size-fits-all campaigns, AI chatbots offer interactive, real-time engagement, making them particularly effective in addressing nuanced health behaviors and misconceptions [16]. The effectiveness of chatbot-based interventions in promoting behavior change is well supported in the literature. For instance, multiple studies have reported high levels of engagement, usability, and satisfaction with chatbot interventions targeting adolescents and adults [16-18]. Recent studies have explored innovative approaches to promote HPV vaccination, particularly among adolescents and young adults. Digital health interventions, including chatbots, have shown promise in improving knowledge and vaccination intentions [19-21]. Particularly, a study conducted in South Korea developed an eHealth communication intervention using a KakaoTalk chatbot, which shows promising usability scores among experts and adolescent girls [20].

Although existing studies demonstrate the potential of chatbots to influence health behaviors through interactive and personalized engagement, there remains a significant gap in culturally tailored chatbot interventions specifically designed for HPV prevention and cancer risk reduction. Despite the growing evidence base, no studies have focused on developing a chatbot that addresses the unique cultural and linguistic barriers faced by specific populations, such as Korean immigrants and Korean Americans.

To address these gaps, we aimed to develop K-Bot, an AI chatbot designed to deliver culturally tailored, bilingual (English and Korean) health information, specifically targeting Korean immigrants and Korean Americans—groups with unique cultural and linguistic barriers to HPV vaccination [8]. This study also aimed to evaluate the usability of K-Bot to assess how easily and effectively the tool can be used as a culturally sensitive, AI-powered chatbot providing real-time health information about HPV vaccination.

## Methods

### Study Design

The development and usability evaluation of K-Bot followed a structured, iterative process comprising 3 phases: chatbot development, expert evaluation, and usability testing. Evaluations were conducted with both a multidisciplinary expert panel and user groups to ensure the chatbot’s clinical relevance, cultural sensitivity, and practical usability.

### Ethical Considerations

All study procedures were approved by the Institutional Review Board (IRB) of the University of Cincinnati (IRB protocol 2022-0347). All study procedures were conducted following institutional guidelines and ethical standards for research involving human participants. Informed consent was obtained from all participants prior to their participation.

### Chatbot Development

The development of the K-Bot chatbot was based on a structured and systematic process leveraging both advanced technology platforms and cultural tailoring to address HPV vaccination disparities among Korean Americans.

#### Platform Selection and Chatbot Design

K-Bot’s development required a multidisciplinary team, including AI developers from South Korea who were integral to the implementation of the AI chatbot and its deployment on relevant platforms. The team also included experienced health researchers in designing and evaluating chatbots from South Korea, 2 health care professionals based in the United States and 2 Korean community representatives, who provided insights to ensure cultural relevance. This diverse team brought together expertise in AI, health care, and cultural adaptation to ensure the chatbot’s relevance and effectiveness across both Korean and Korean American populations.

**Figure 1:**
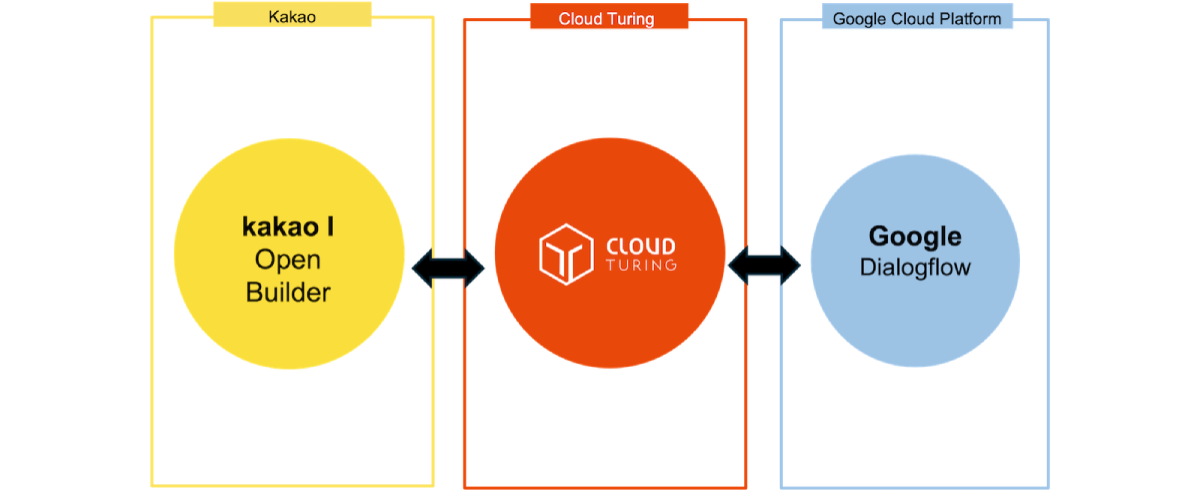
K-Bot architecture and integration with the existing management system.

The chatbot was built using CloudTuring [22], a platform offering AI-driven solutions for advanced natural language understanding, and Google Dialogflow [23], a widely used conversational AI framework that enables natural language processing (NLP), intent recognition, and context-aware responses. The chatbot was integrated with the existing management system, allowing for continuous updates and improvement through the Kakao Chatbot Manager and Cloud Turing platforms (Figure 1).

**Figure 2:**
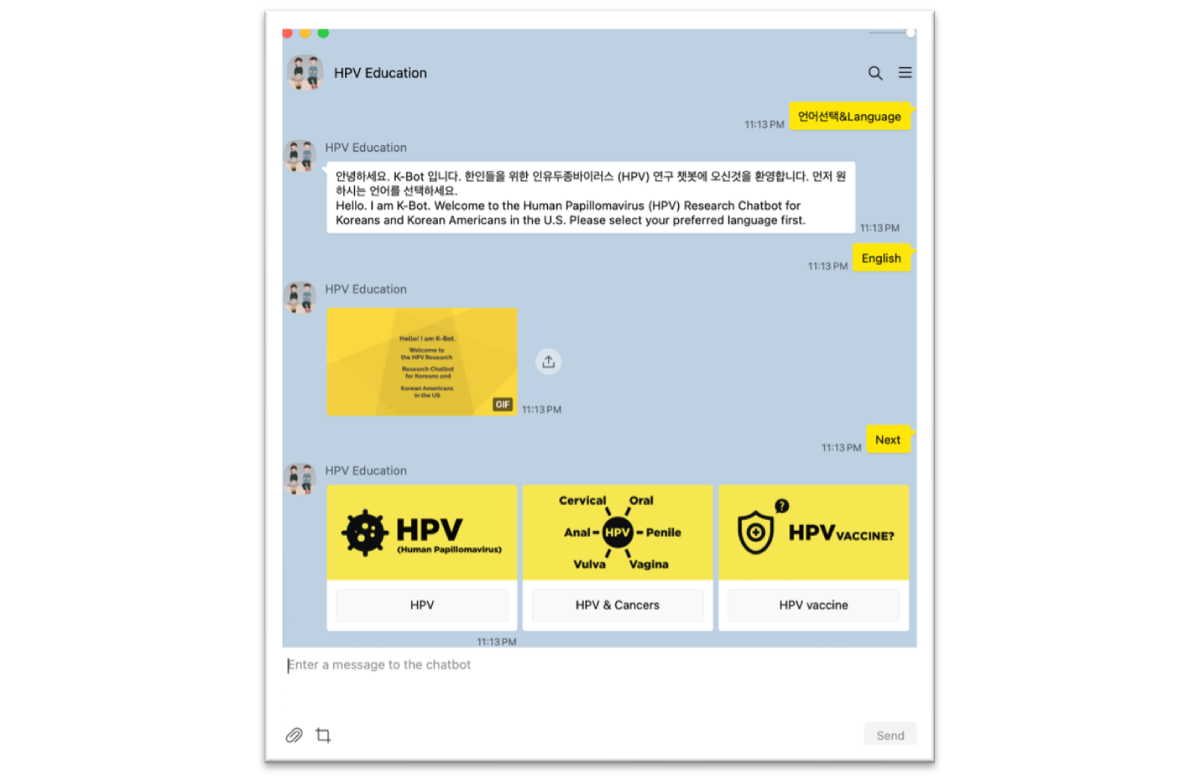
K-Bot interface within the KakaoTalk app. HPV: human papillomavirus.

These tools facilitated bilingual interactions in English and Korean, supporting accurate communication and seamless user engagement. Deployment was carried out on the KakaoTalk messaging app (Figure 2), which is widely used by Koreans for communication [24], and a Wix-based website, requiring secure hosting and compliance with privacy standards. The development process spanned 5 months, with iterative refinements made based on expert and user feedback.

The chatbot’s dialogues were designed based on evidence-based guidelines from the Centers for Disease Control and Prevention (CDC) and tailored to the Korean American population using findings from previous studies [12,25,26].

The chatbot was structured using a hierarchical organization of major, medium, and minor classifications, which guided the users toward their desired information. Each interaction was categorized under major topics (eg, HPV vaccine), followed by medium-level subcategories (eg, vaccine safety and side effects), and further segmented into minor classifications (eg, detailed explanations of side effects). This modular structure ensured logical navigation and efficient access to information, which is critical for users seeking health-related information.

#### Structured Evaluation and Refinement Process

**Figure 3:**
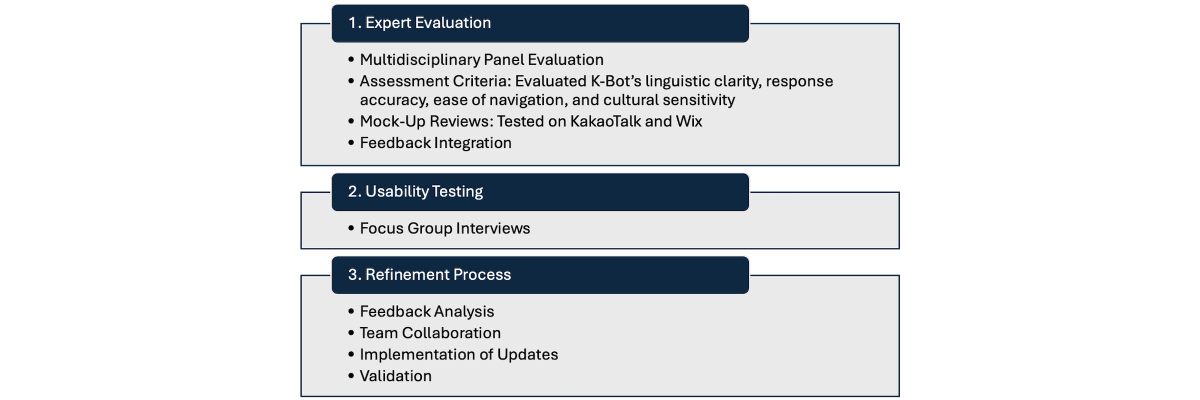
Evaluation and refinement process of K-Bot.

The evaluation and refinement of K-Bot followed 3 distinct phases: (1) expert evaluation, (2) usability testing, and (3) refinement (Figure 3).

##### Expert Evaluation

A multidisciplinary panel conducted a comprehensive evaluation of K-Bot, leveraging expertise in health care, cultural studies, AI, and community engagement. The panel included a clinician specializing in HPV prevention, researchers with extensive knowledge of Korean culture and its influence on health behaviors, an AI specialist experienced in chatbot development, and students in health-related fields representing Korean community perspectives. This diverse composition enabled a holistic assessment, ensuring the chatbot’s clinical relevance, cultural sensitivity, technical functionality, and alignment with community needs.

The evaluation process systematically assessed K-Bot’s responses, navigation, and cultural appropriateness using predefined criteria. These criteria included linguistic clarity, response accuracy, ease of navigation, and the cultural framing of sensitive topics, such as HPV vaccination. The usability evaluation framework incorporated principles from the International Organization for Standardization (ISO) 9241-11 standards, emphasizing efficiency, effectiveness, and user satisfaction to ensure comprehensive assessment [27]. The chatbot’s response design followed a structured methodology that incorporated synonyms, key phrases, and importance ratings on a scale of 1-5, with 5 indicating the highest priority for delivering critical information. Experts rated responses based on their relevance, clarity, and alignment with the cultural and informational needs of the target audience, ensuring the chatbot provided accurate and contextually appropriate replies.

The research team conducted mock-up reviews to evaluate the chatbot before usability testing. One review used the English version, and the other used the Korean version of the chatbot. Additionally, the team tested the chatbot on 2 different platforms: KakaoTalk and Wix. These sessions focused on identifying areas for improvement, such as simplifying technical terms, integrating culturally relevant idioms, and enhancing navigation flow to optimize the user experience before moving forward with usability testing involving participants.

Iterative improvements based on the research team’s feedback were incorporated into the chatbot’s design, prioritizing culturally sensitive topics and user-relevant queries. Linguistic clarity was enhanced by simplifying technical terms and integrating culturally relevant idioms to increase accessibility. These revisions were discussed and validated during research team meetings, confirming improvements in usability, cultural sensitivity, and alignment with the needs of Korean immigrants and Korean American users.

##### Usability Testing

###### Recruitment and Eligibility

Participants were recruited using a combination of the researchers’ networks, a community-engaged approach, and social media platforms guided by the previous successful recruitment [6]. Recruitment efforts were centered around the research theme, “Let’s Talk About HPV,” which was designed to engage participants and emphasize the importance of learning about HPV and its vaccine. Potential participants who expressed interest in the study were contacted within 1 week by research personnel to confirm their eligibility. Eligibility criteria included (1) individuals aged 18-45 years, (2) possession of a mobile phone or a computer with internet access, (3) residence in the United States, and (4) fluency in English or Korean. Upon confirming eligibility, research personnel scheduled an interview and provided participants with a password-protected Zoom link via email.

###### Usability Testing Procedures

Usability testing was conducted between May and June 2022, involving 21 Korean American participants who were divided into 6 focus groups, with each group consisting of 2-5 individuals. The variation in group size allowed us to accommodate participants’ availability and facilitate in-depth discussions. Data saturation was monitored by the facilitators throughout the sessions. Each focus group session lasted approximately 60-90 minutes. Only audio recordings were used for data analysis, ensuring that participants’ identities remained confidential. Participants were encouraged to use pseudonyms instead of real names to promote anonymity. The primary objective of this study was to gather feedback on the K-Bot rather than to explore participants’ awareness and attitudes toward HPV prevention. Therefore, the sensitive nature of HPV-related questions was minimal. Participants were informed about the purpose of the study, the voluntary nature of participation, and their right to withdraw at any time without consequence.

Each usability testing session followed a structured protocol to ensure consistency and comprehensive data collection:

- Introduction: Participants were introduced to the study, its objectives, and the importance of their feedback in improving the chatbot’s functionality and cultural relevance. The project’s development history, including initial focus on Korean college women and subsequent expansion to a broader age range, including both men and women, was explained.
- Consent and survey: After reviewing the study information sheet and agreeing to participate, participants were provided with an online survey. This survey collected information about their sociodemographic characteristics, awareness of HPV, the HPV vaccine, and their vaccination status.
- Preinteraction discussion: Prior to interacting with K-Bot, participants were asked questions regarding their familiarity with HPV-related topics and any previous experience with chatbots. Specifically, we discussed whether they had heard of HPV or its vaccine and whether they believe they had received adequate information about the topic.
- Chatbot interaction: After completing the survey, participants received a link via email to interact with K-Bot. They were reminded that the chatbot was available in both English and Korean languages. At this stage of development, the K-bot experience consisted of 2 parts: a 32 question-and-answer (Q&A)–style survey, followed by the interactive chatbot. Participants were encouraged to document any errors, navigation challenges, or suggestions for improvement during their interaction. Additionally, K-Bot provided links to external resources, such as CDC PDF files, to test its interoperability and ensure seamless access to supplementary health information.
- Postinteraction feedback: We asked participants questions guided by Peter Morville’s honeycomb model [27], as shown in Table 1. Participants were asked about usability, ease of navigation, adequacy of information, reliability of chatbot responses, and integration with external resources. They also provided suggestions for improvement. After the interview, participants received a US $20 Amazon gift card as compensation for their time and input.

**Table 1:** Semistructured interview questions with Morville’s usability framework.

Usability dimension	Definition	Corresponding interview questions
Usability	Ensures the system is easy to use and navigate	How was your experience with K-Bot? Did you find it easy? Were the buttons and navigation clear and easy to understand?
Usefulness	Determines whether the system provides valuable and relevant content	Was there enough information provided by the chatbot?
Credibility	Evaluates the reliability and trustworthiness of the system’s responses	Were the responses from the chatbot reliable?
Findability	Assesses the ease of accessing information within the system	Was it interoperable with external sources? Were the PDF files for the CDCa resource pages loading and adequate?
Desirability	Examines the appeal and attractiveness of the system design	What did you think about the overall design of the chatbot? Did you find the chatbot visually appealing and engaging?
Accessibility	Ensures the system is available and usable for a diverse audience	Was there any information you wish was included in the chatbot?
General feedback	Captures any additional participant input for further refinement	Any additional comments?

###### Data Collection and Analysis

An online survey that takes 5-10 minutes was conducted prior to qualitative interviews to gather insights into participants’ demographics (eg, age, sex, cultural identification, generational status, educational attainment, marital status, employment status, and annual household income), HPV awareness, and vaccination status. Quantitative data were analyzed using descriptive statistics with SPSS version 28 (IBM Corp). All focus group sessions were recorded using Zoom, and audio recordings were captured via Audacity [28] as backup. Transcriptions were manually prepared and saved in Microsoft Excel, with anonymized participant IDs to maintain confidentiality. We monitored the duration of participants’ interactions with K-Bot, which typically lasted about 10-15 minutes. All data were stored securely on password-protected drives.

Qualitative data were manually analyzed using a systematic thematic analysis to identify recurring patterns in feedback. The analysis followed Braun and Clarke’s 6-phase framework: familiarization with data, generating initial codes, searching for themes, reviewing themes, defining and naming themes, and producing a report [29]. Transcripts were independently coded, guided by 2 researchers using Morville’s usability framework, covering aspects such as usefulness, usability, findability, credibility, accessibility, desirability, and value. Coding discrepancies were resolved through discussion to ensure a systematic approach.

##### Refinement

The refinement process was structured to systematically integrate insights from usability testing into iterative improvements into the chatbot’s design and functionality. Feedback collected during usability sessions was reviewed and categorized into thematic areas, including navigation, content clarity, and technical performance.

- Feedback analysis: All participant suggestions, navigation issues, and identified gaps were documented and categorized. For example, repeated difficulties with navigating back to previous sections were flagged as high-priority issues.
- Team collaboration: Key findings were shared with the chatbot development team during research team meetings. Actionable items were prioritized based on their potential impact on user experience and alignment with study objectives.
- Implementation of updates: Adjustments were implemented through CloudTuring. Navigation was improved by adding clearer button labels and a dedicated Help section to guide users. The content was expanded to include additional HPV-related information that participants indicated as missing. Machine learning algorithms were updated to improve response accuracy and ensure that the chatbot delivered contextually relevant answers.
- Validation: Updates were tested internally by the research team to ensure that changes effectively addressed the identified issues, contributing to the dependability and confirmability of the intervention’s iterative development.

## Results

### Quantitative Findings

#### Sociocultural Demographic Characteristics

As presented in Table 2, participants had a mean age of 23.67 (SD 4.66) years. The majority were female (12/21, 57.1%), second-generation individuals (13/21, 61.9%), and single (20/21, 95.2%).

**Table 2:** Demographic characteristics of focus group participants (N=21).

Characteristics		Value
Age (years; range 18-37), mean (SD)		23.67 (4.66)
**Sex, n (%)**		
	Female	12 (57.1)
	Male	9 (42.9)
**Cultural identification, n (%)**		
	Completely or more Korean than American	6 (28.5)
	Equally Korean and American	9 (42.9)
	More American than Korean	6 (28.6)
**Generational status, n (%)**		
	First generation (born outside the United States)	8 (38.1)
	Second generation (born in the United States)	13 (61.9)
**Education, n (%)**		
	High school degree or equivalent	6 (28.6)
	Some college, no degree	3 (14.3)
	Bachelor’s degree	9 (42.9)
	Master’s degree	3 (14.3)
**Marital status, n (%)**		
	Single (never married)	20 (95.2)
	Married or in a domestic partnership	1 (4.8)
**Current employment status, n (%)**		
	Full time (≥40 hours)	9 (42.9)
	Part time (≤39 hours)	2 (9.5)
	Student	10 (47.6)
**Annual household income (US $), n (%)**		
	<50,000	8 (38.1)
	50,000-99,999	8 (38.1)
	≥100,000	5 (23.8)

#### Awareness of HPV and HPV Vaccination

As presented in Table 3, most participants had heard of HPV (19/21, 90.5%) and the HPV vaccine (18/21, 81.8%). Half (11/21, 52.4%) of them had received the HPV vaccine, with 7 (63.6%) of the 11 participants completing all 3 doses.

**Table 3:** HPV^a^ awareness and vaccination status of focus group participants by sex (N=21).

Characteristics			Female (n=12) frequency, n (%)	Male (n=9) frequency, n (%)
**Heard of HPV before this study**				
	Yes	11 (91.7)		8 (88.9)
	No	1 (8.3)		1 (11.1)
**Heard of HPV vaccine or Gardasil before this study**				
	Yes	11 (91.7)		7 (77.8)
	No	1 (8.3)		2 (22.2)
**Received the HPV vaccine**				
	Yes	7 (58.3)		4 (44.4)
	No	0		3 (33.3)
	Do not know	5 (41.7)		2 (22.2)
**Number of HPV vaccine doses received** (n=11)				
	1	0		1 (25.0)
	2	2 (28.6)		1 (25.0)
	3	5 (71.4)		2 (50.0)

^a^HPV: human papillomavirus.

### Qualitative Findings and Refinement

Based on the preinteraction discussion and postinteraction feedback, participants highlighted the importance of K-Bot’s bilingual functionality, expressing appreciation for its ability to deliver information in both English and Korean. Many found the information about HPV and the HPV vaccine to be helpful, with some sharing that they learned new details they were previously unaware of. Some participants who had completed middle and high school in Korea had never heard of HPV and its vaccine. Some participants also shared they came in with the preconception that the vaccine was primarily for women’s health. However, after interacting with the chatbot, 1 (4.8%) female participant stated, “I feel like I would want my spouse, or partner, to have it.” One male participant mentioned that he thought he would not be affected by HPV, but the interactions with K-Bot “put it into perspective that maybe I’m also prone to it.” This positive change in sentiment toward the HPV vaccine was shared by other male participants in the focus groups.

The usability of the K-Bot intervention was evaluated through focus group interviews guided by Morville’s usability framework:

- Usability: Focus group participants found that the Q&A format at the beginning of the interaction was time-consuming as some “stopped reading the info after answering the question” and found it “cumbersome.” The Q&A section was removed and consolidated into the interactive portion. However, participants found the chatbot’s button-based Q&A format simple and intuitive, with 1 (4.8%) participant mentioning it was like a “very guided Q&A session.” Several requested more detailed information about complex topics, such as stress and HPV. To address this, expandable sections were introduced into the interactive K-Bot, allowing users to delve deeper into specific topics, while maintaining the simplicity of the button-based structure.
- Usefulness: External links to resources were valued for their credibility, but broken links were noted as a barrier to usability. All external links were repaired, and additional resources, including directories of health care providers, were added to ensure comprehensive support. Tailored resources were also developed to meet the needs of specific demographics, particularly first-generation Korean Americans. For instance, 1 (4.8%) participant voiced that “it would be helpful if you add the term [for HPV] that is used more generally in Korea” since they may not recognize the term “human papilloma virus” or “HPV.”
- Credibility: The credibility of the chatbot was bolstered by its evidence-based content and integration with trusted sources, such as the CDC. However, participants emphasized the importance of culturally relevant information tailored to Korean Americans, especially for those more familiar with Korean resources. Automatic PDF file downloads were replaced with direct links to reputable sources as participants did not like “surprise downloads crowding the phone.” This change ensured easier access, while maintaining trust, especially with concerns about the possibility of users not “trusting the information on the K-Bot.” Additionally, culturally sensitive content and terms were refined to resonate with the target demographic.
- Findability: Participants appreciated the structured menus but reported difficulties navigating the menu items at the medium and minor classifications. For instance, 1 (4.8%) participant remarked, “I kept forgetting what I read versus what I did not read, because there were so many subcategories.” To resolve this, the final menu options were simplified through consolidation of similar topics, and a search function was added to help users quickly locate relevant information. Visual cues, such as highlighted buttons and GIFs, were also incorporated to guide users more effectively through the content.
- Desirability: To increase desirability, participants suggested incorporating visual elements to improve engagement and support users with varying health literacy. In response, GIFs and images were added to the chatbot interface. One participant commented, “The menu items were clear but could use more explanations for people who are not familiar with medical terms.” Additional descriptions were added to clarify technical terms, and a “Back” button was introduced to enable users to revisit previously accessed information, enhancing overall navigation.
- Accessibility: Although participants found the chatbot intuitive, some noted challenges with language switching and navigation at the end of interactions. For example, 1 (4.8%) participant shared, “It was not immediately clear how to switch languages at the beginning.” To address this, clearer instructions for language switching were integrated during onboarding, and the navigation flow was streamlined to ensure smooth transitions, particularly during extended interactions. Button formats were also standardized for greater usability.

Feedback from participants provided valuable insights into their interaction with the chatbot and informed specific refinements to enhance its effectiveness (Table 4). Key refinements were implemented based on usability testing to address the needs of Korean immigrants and Korean American users and optimize the intervention for promoting HPV vaccination.

**Table 4:** Findings from semistructured interviews and refinements.

Usability dimension	Findings	How the findings were addressed
Usability	The button-based navigation was intuitive. Participants suggested adding a “Back” button. Some users were confused by the language selection.	A “Back” button was introduced to enable participants to revisit previously accessed information. Language-switching instructions were made clearer and added as part of the onboarding.
Usefulness	Participants valued information about HPV^a^ transmission and vaccination. External links (eg, CDC^b^ fact sheets) were credible and useful. Suggestions included using visuals to reduce cognitive load.	Animated GIFs were integrated to complement text-heavy content. External links were checked, repaired, and updated. Resources were tailored to specific demographics, especially first-generation Korean Americans.
Credibility	Evidence-based content and links to trusted sources enhanced credibility. Feedback emphasized culturally sensitive language and early trust building.	Automatic PDF file downloads were replaced with direct links to trusted sources, such as the CDC. Culturally relevant terms and tailored content for Korean Americans were refined. An introductory message established credibility and outlined the chatbot’s purpose.
Findability	Menus were structured and effective but sometimes overwhelming. A simplified navigation structure was recommended.	The final menu options were simplified, and a search function was added to help users quickly locate relevant information. Visual cues, such as highlighted buttons and GIFs, were incorporated to improve findability and guide users effectively.
Desirability	Clear icons and buttons were appreciated. Suggestions were made for animations and introduction videos to enhance engagement.	Visual elements, including GIFs and images, were introduced to engage users. Animations and a 2-minute introduction video were included to enhance user appeal.
Accessibility	Bilingual support in English and Korean was well received. Broken links hindered accessibility; regular updates were suggested.	An email address for the bilingual researcher was added to allow participants to report any issues with broken links or accessibility.
General feedback	Suggestions for demographic-specific content, clearer instructions, and interactive features were provided. Misconceptions about the HPV vaccine being unnecessary for men were corrected. Participants realized the importance of vaccinating at a young age.	Demographic-specific resources and culturally sensitive information were added. A brief instruction page and visual cues were included to guide users through the chatbot. The culturally tailored K-Bot fostered a more open and informed dialogue, centered around increasing the awareness and understanding of HPV.

^a^HPV: human papillomavirus.

^b^CDC: Centers for Disease Control and Prevention.

## Discussion

### Principal Findings

This study examined the development and usability evaluation of K-Bot, an AI chatbot created to provide bilingual, culturally sensitive health information about HPV vaccination for Korean immigrants and Korean Americans. The study assessed the chatbot’s usability in delivering accurate health information and identified both strengths and areas for refinement, contributing to the optimization of chatbot-based health interventions aimed at promoting behavior change and informing the development of more targeted and effective strategies in future implementation trials.

This study found that although participants exhibited high awareness of HPV and its vaccine, actual vaccine uptake remained low, particularly among male participants. Participants reported that K-Bot addressed this disparity by providing inclusive education, which is critical for promoting vaccine uptake among all individuals. Participants identified K-Bot’s bilingual functionality and culturally tailored content as significant strengths, with the dual-language option allowing access to health information in both English and Korean. This finding highlights the necessity of linguistic inclusivity in digital health tools to engage diverse populations effectively. However, the usability evaluation revealed areas for improvement, including navigation complexity, challenges with language selection, and limited visual engagement. Although this study incorporated GIFs and images to improve visual engagement and support users with varying levels of health literacy, these enhancements alone may be insufficient to address the broader and more nuanced challenges faced by diverse user populations. Addressing these limitations through iterative design refinements and leveraging emerging technologies, such as multimodal conversational systems, could further optimize K-Bot’s usability and impact on health behavior change.

### Comparison With Prior Work

The findings of this study align with the existing literature, indicating that HPV vaccination is often perceived as primarily relevant to women, particularly within the Korean community [12,25,30]. Previous research has shown that cultural perceptions and public health messaging in Korea have historically targeted females, contributing to persistent disparities in vaccination rates by sex [31]. This misconception can be traced back to Korea’s initial HPV vaccination program, which was launched in 2016, focusing exclusively on adolescent girls before expanding to include boys in 2024 [32]. This focus on vaccinating only specific groups likely influenced public attitudes among Koreans and Korean Americans, as immigrant health behaviors are often shaped by practices from their country of origin [12,25]. This exclusion of boys may have perpetuated misconceptions in Korean American communities, furthering barriers to vaccine uptake, particularly where familial and community norms strongly influence health decisions, [33]. Similar findings in previous studies suggest that these culturally ingrained beliefs can create significant barriers to HPV vaccination among males, necessitating targeted educational intervention [12]. Addressing these challenges requires inclusive education tailored to Korean Americans, which highlights the importance of HPV vaccination for all individuals in preventing disease and reducing transmission. Participants shared that the chatbot helped correct the belief that HPV vaccination is exclusively for women, a misconception that has been widely documented in the literature as a significant barrier to male vaccination [34]. Culturally tailored digital interventions, such as K-Bot, have the potential to play a critical role in addressing these challenges.

K-Bot’s culturally tailored approach aligns with a growing body of research advocating for the use of culturally sensitive design elements. Studies have shown that culturally sensitive design elements, such as language adaptability, empathy, and humor, enhance user trust, engagement, and the overall efficacy of digital health interventions [35]. By integrating a culturally sensitive design, K-bot exemplifies the potential of AI-driven technologies to mitigate health care disparities by improving access to accurate, culturally relevant health information. The study also adds to the emerging evidence on the existing literature focusing on culturally tailored digital interventions in mitigating barriers to HPV vaccination. Although the domain of culturally adapted chatbots remains underexplored, this study reinforces the critical need for such interventions to address cultural stigmas and misconceptions surrounding HPV. The existing literature highlights the critical need for culturally sensitive interventions, especially given the deeply rooted cultural stigmas and misconceptions surrounding STIs in many Asian American communities [36,37]. These stigmas often act as significant deterrents, discouraging open discussions about HPV vaccination, particularly among Asian immigrant populations where cultural norms may further limit access to accurate health information [37]. By directly addressing these cultural and linguistic barriers, K-Bot represents an important advancement in leveraging AI-driven tools to deliver tailored health information and promote equitable access to preventative care.

### Strengths and Limitations

This study’s strengths lie in its rigorous, iterative design process, combining expert evaluation with participatory user feedback to optimize K-Bot’s functionality. Integrating user feedback into iterative refinements follows the best practices in participatory design for digital health interventions [37], emphasizing the importance of collaborative frameworks in enhancing usability and user satisfaction.

This study has several limitations that should be considered when interpreting the findings. First, the limited sample size reduces the generalizability of the results, as the insights gained may not fully represent the broader population. Second, although K-Bot’s culturally tailored design was well received by this specific group, its applicability to other Asian American subgroups or culturally diverse populations remains uncertain. Lastly, the study primarily relied on self-reported data, introducing potential biases, such as social desirability and recall bias, which may affect the accuracy of the findings.

### Future Research Directions

The findings underscore K-Bot’s potential as both a stand-alone intervention and an integrated tool within health care systems to address HPV vaccination disparities. Future research should focus on evaluating its feasibility and efficacy through rigorous trials, with particular attention to its scalability across diverse health care settings. Longitudinal studies are essential to assess K-Bot’s sustained impact on vaccination uptake and behavior change. Expanding K-Bot to other Asian American subgroups would enhance its adaptability and engagement, addressing the heterogeneity within these populations. K-Bot’s reliance on NLP, while effective in delivering structured and evidence-based information, presents limitations in managing open-ended queries. K-Bot was developed prior to the emergence of generative AI technologies, such as Chat Generative Pretrained Transformer (ChatGPT). The recent literature examining the application of ChatGPT in nursing education, practice, and research highlights several advantages of generative AI [38]. These capabilities suggest significant potential for enhancing K-Bot’s functionality by enabling more dynamic, context-sensitive, and user-centered interactions. However, as noted in the literature, ethical considerations, such as minimizing misinformation and ensuring cultural sensitivity, must be prioritized to safeguard the intervention’s credibility and trustworthiness [38]. Although K-Bot’s reliance on NLP ensures accurate, evidence-based responses, its limited ability to manage open-ended queries highlights a need for integrating advanced AI technologies to better meet user needs. Additionally, usability feedback from this study may differ among various age groups, with younger users potentially being more tech-savvy than older adults. Future iterations of K-Bot could explore tailoring its interface and functionality to accommodate different subgroups, such as adolescents and parents, to enhance user engagement and satisfaction.

### Conclusion

In conclusion, K-Bot exemplifies an innovative, culturally sensitive approach to addressing HPV vaccination disparities among Korean Americans. By delivering personalized, bilingual health information, K-Bot has successfully demonstrated its potential to overcome barriers related to language, stigma, and misinformation. Although the study’s findings are encouraging, they are based on a smaller sample size, and further research is needed to validate these results across a broader and more diverse population. Refinement of the chatbot’s usability and potential customization for different age groups and cultural contexts could enhance its effectiveness. The positive reception of K-Bot suggests it could be a scalable tool for public health interventions. Exploring the application of AI-driven chatbots in other populations remains a promising avenue for addressing health inequities and promoting equitable health care access.

## Abbreviations

ACIP: Advisory Committee on Immunization Practices
AI: artificial intelligence
CDC: Centers for Disease Control and Prevention
ChatGPT: Chat Generative Pretrained Transformer
HPV: human papillomavirus
NLP: natural language processing
Q&A: question-and-answer
STI: sexually transmitted infection

## Data Availability

Data are presented in the main manuscript.
